# Supplementary material for: Capturing continuous, long timescale behavioral changes in Drosophila melanogaster postural data
Source: ArXiv. 2023 Sep 7:arXiv:2309.04044v1. Preprint. [Version 1] (PMC10508836)
Supplement: 1 [file NIHPP2309.04044V1-supplement-1.pdf]

Supplementary Information

Tables

Date	Experiment	Camera	Start Time (UTC)	File
2/17/2022	exp1	Camera 1	2022/02/17 17:37:18	20220217-lts-cam1
2/17/2022	exp1	Camera 2	2022/02/17 17:37:18	20220217-lts-cam2
2/17/2022	exp1	Camera 3	2022/02/17 17:46:39	20220217-lts-cam3
2/17/2022	exp1	Camera 4	2022/02/17 17:46:39	20220217-lts-cam4
3/13/2022	exp2	Camera 3	2022/03/13 02:14:15	20220313-lts-cam3
3/13/2022	exp2	Camera 4	2022/03/13 02:14:15	20220313-lts-cam4
3/26/2022	exp3	Camera 3	2022/03/26 19:50:45	20220326-lts-cam3
3/26/2022	exp3	Camera 4	2022/03/26 19:50:45	20220326-lts-cam4
4/18/2022	exp4	Camera 1	2022/04/18 19:06:00	20220418-lts-cam1
4/18/2022	exp4	Camera 2	2022/04/18 19:06:00	20220418-lts-cam2
4/18/2022	exp4	Camera 3	2022/04/18 19:07:28	20220418-lts-cam3
4/18/2022	exp4	Camera 4	2022/04/18 19:07:28	20220418-lts-cam4

Table 1. Metadata table outlining the data collected. Each row represents a single camera recording on a single day covering 4 wells. Here, we show the composition of experiments starting from mid day to evening. The provided File column corresponds to the H5 file containing tracks of each recording. Complete metadata at the per-fly level is provided in the associated data repository.

Figures

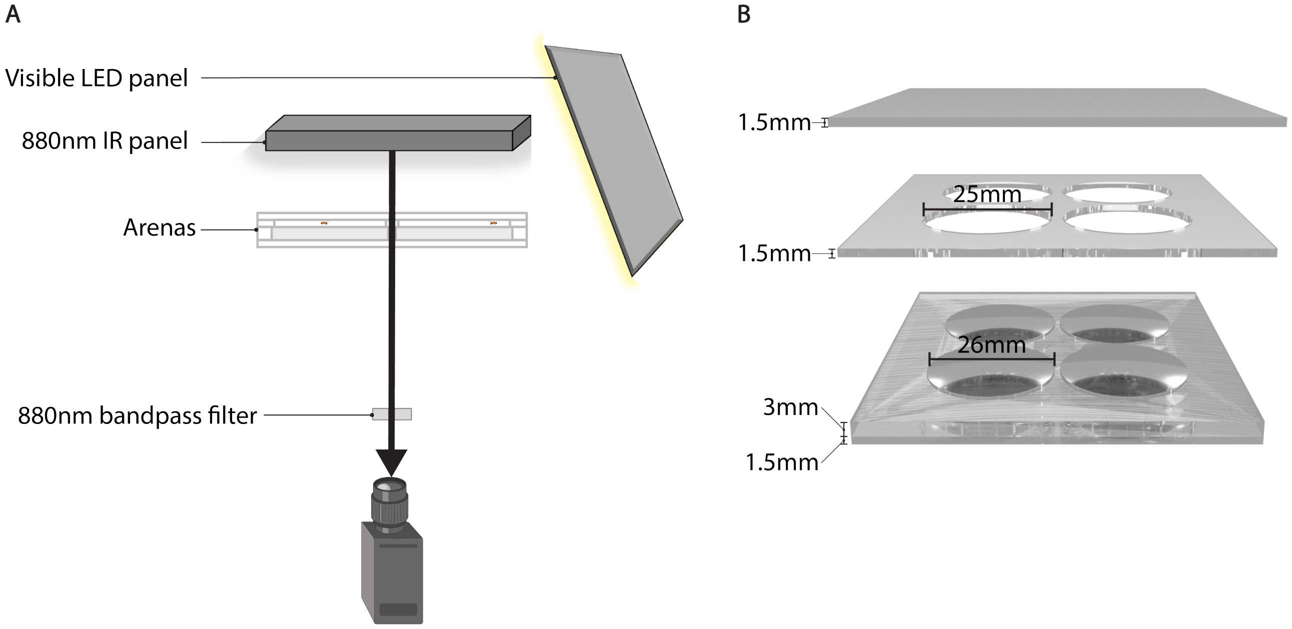

Figure S1. A Schematic of imaging setup. Experimental arenas were illuminated from above with 880nm LED pads to permit constant recording and two visible LED panels (only one shown) on a 12-hour light/dark cycle. Flies were recorded from below using 880nm bandpass filters on each camera to ensure uniformity across visible light changes. **B** Experimental arena schematic. Arenas were constructed from layers of transparent laser-cut acrylic, with a 3mm deep pad of sucrose-agarose media beneath a 1.5mm deep chamber enclosed by a solid layer of acrylic. Arena layers were held together by lab tape, which prevented escape while also permitting airflow.

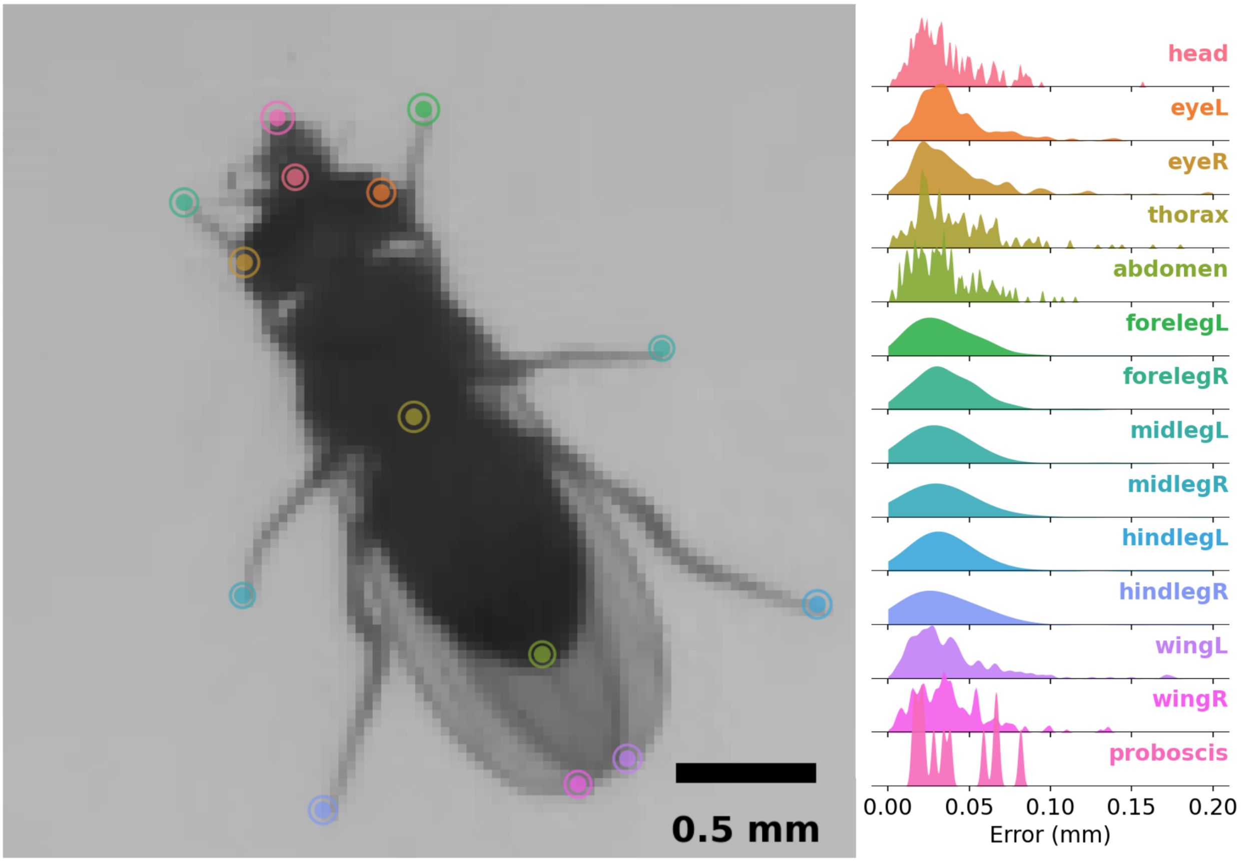

Figure S2. Prediction error plot. The average error distance is approximately 2.22px, corresponding to 78.5µm. Our model's mean average precision (mAP) is 0.70, and the error distance 95th percentile is 4.14px. More metrics and complete models are available in the main dataset.

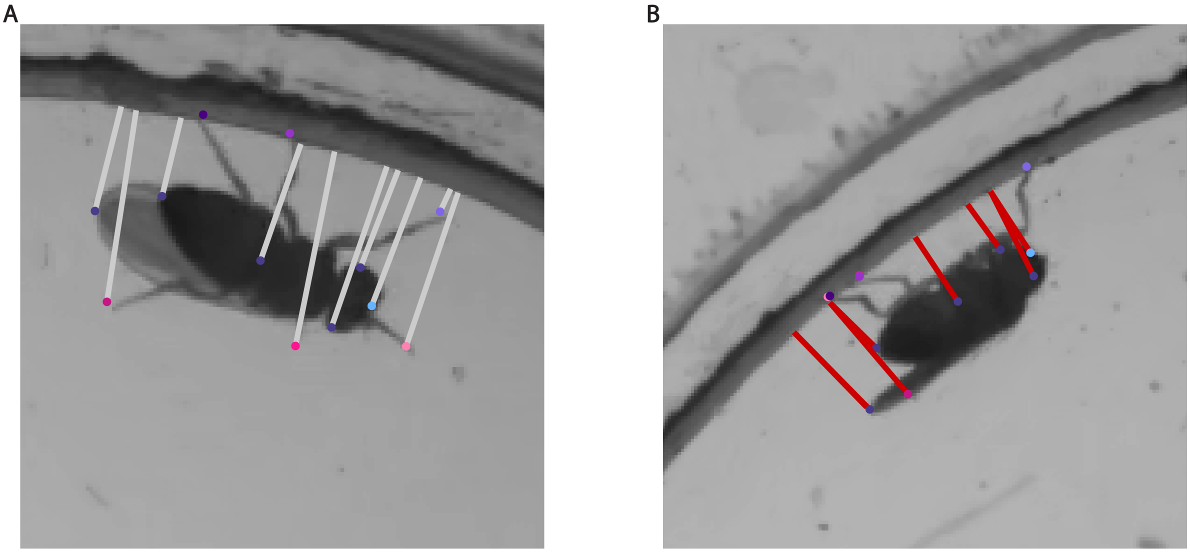

Figure S3. Illustration of edge detection method. An SVM classifier uses the all-by-all distances between all body part coordinates, except for the proboscis, the speed of each body part, and the distance for each body part from the edge to classify time points as 'off edge' (example shown on left) or 'on edge' (example shown on right). Points directly on the edge, such as some of the tarsi in these images, have an edge distance of 0, which naturally cannot be shown.

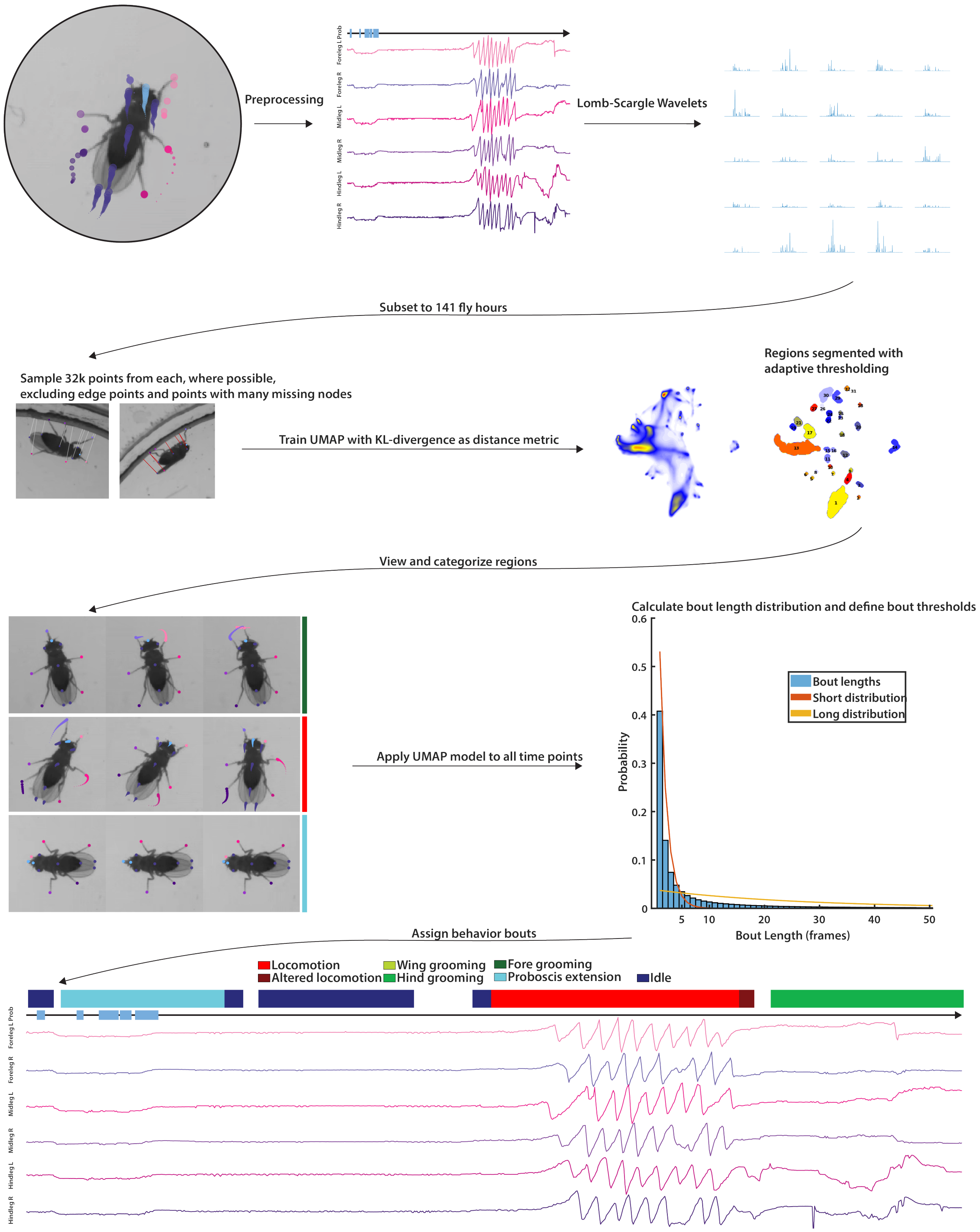

Figure S4. Schematic of behavioral classification pipeline. The behavioral pipeline shows the flow of data from pose estimation from SLEAP through behavioral bout assignment.

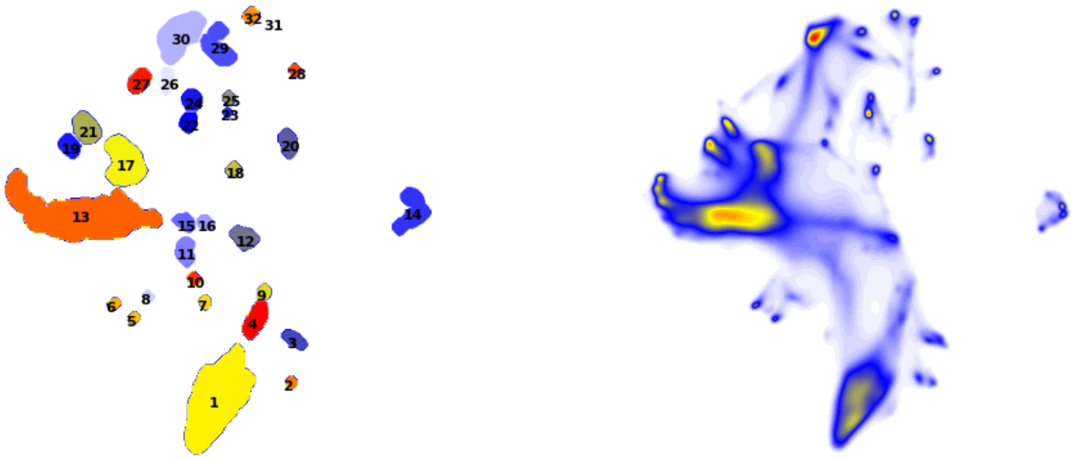

Figure S5. Plot showing the density map of 2D embedding values from UMAP along with the region assignments. Regions 2, 3, 4, 5, 9, 12, 15, 16, 18, 20, 23, 25, 28, 31, and 32 were assigned to idle behavior, 14 was assigned to proboscis extension. Regions 1, 6, 7, 8, 10, and 11 were assigned to foreleg grooming. Regions 26, 27, and 30 were assigned to hind grooming. Regions 22, 24, and 29 were assigned to wing grooming behavior. Regions 17, 19, and 21 were assigned to altered locomotion. Finally, region 13 was assigned to locomotion.

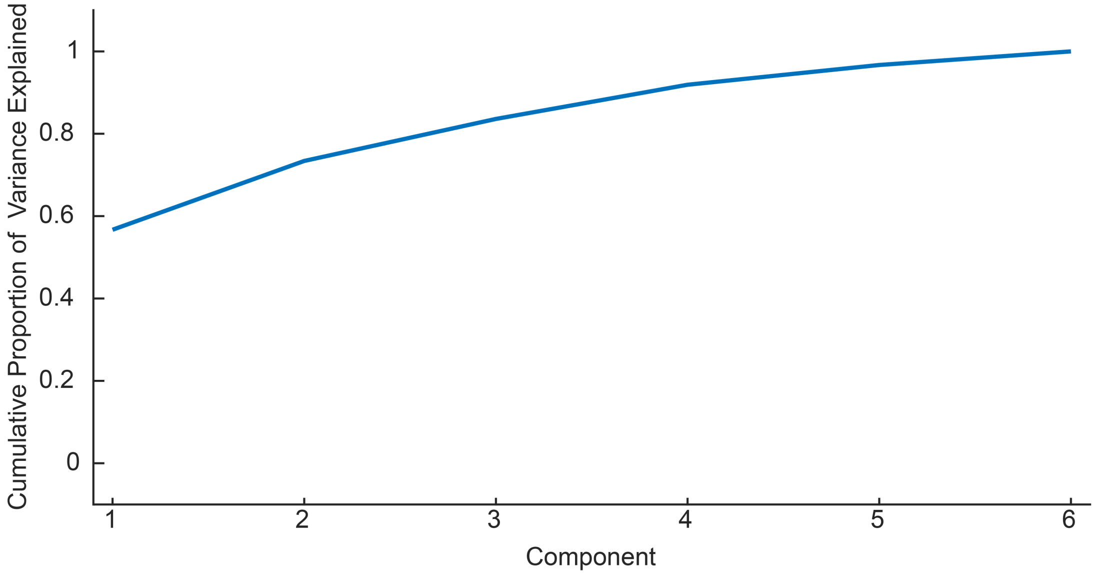

Figure S6. Line plot showing the cumulative variance explained by PCs of behavioral components on the complete data set.

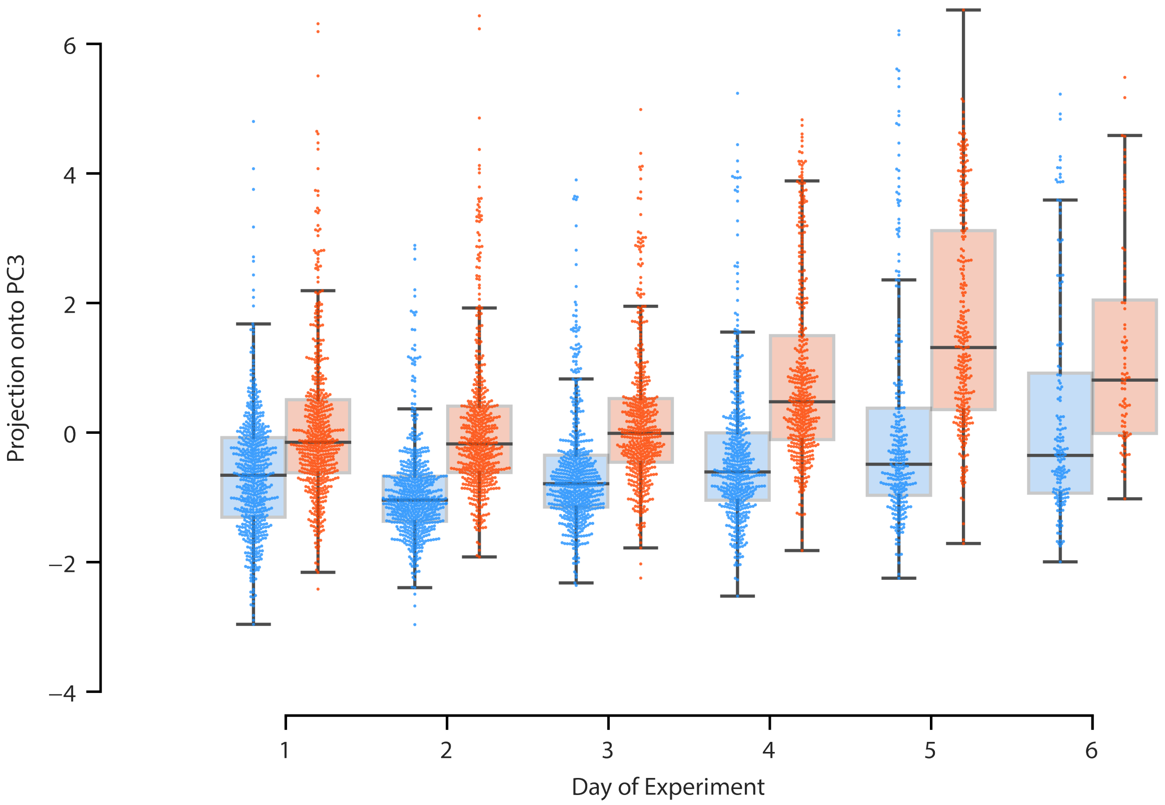

Figure S7. Box plots showing PC3 of behavioral components PCA. Experimental group 1 (experiments 1 and 2) is shown in blue and experimental group 2 (experiments 3 and 4) are shown in red. PC3 separates our experimental groups and this separation becomes more significant farther into the experiments.

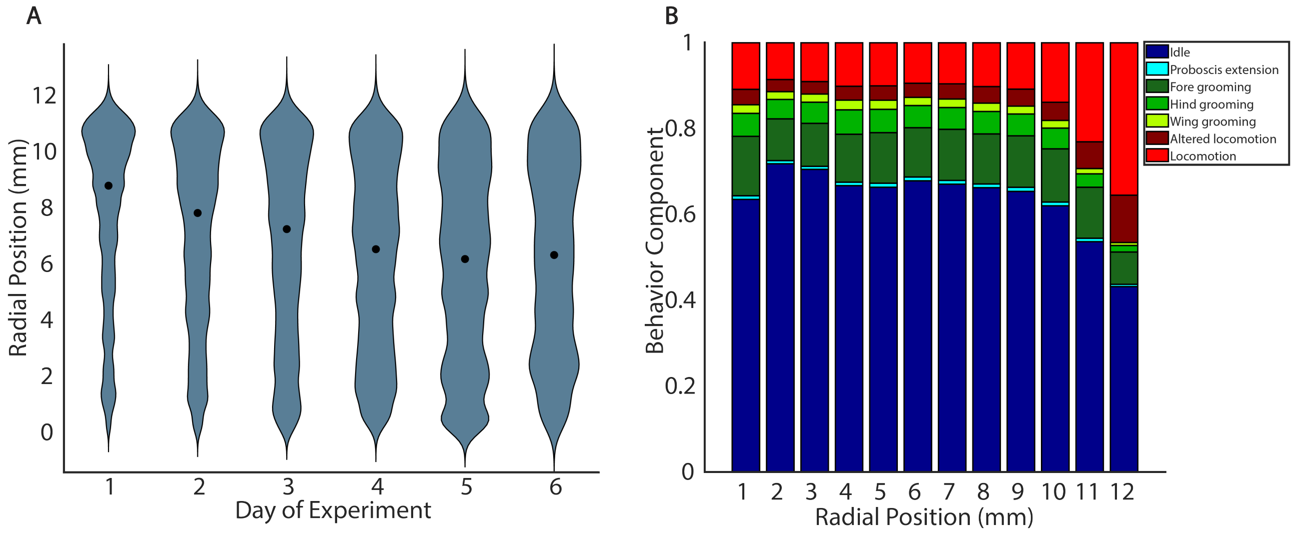

Figure S8. Behavioral characteristics by radial position and radial position distributions by day of experiment. **A** Violin plot showing the distributions of radial position by day of experiment. **B** Barplot of behavioral components by radial position.

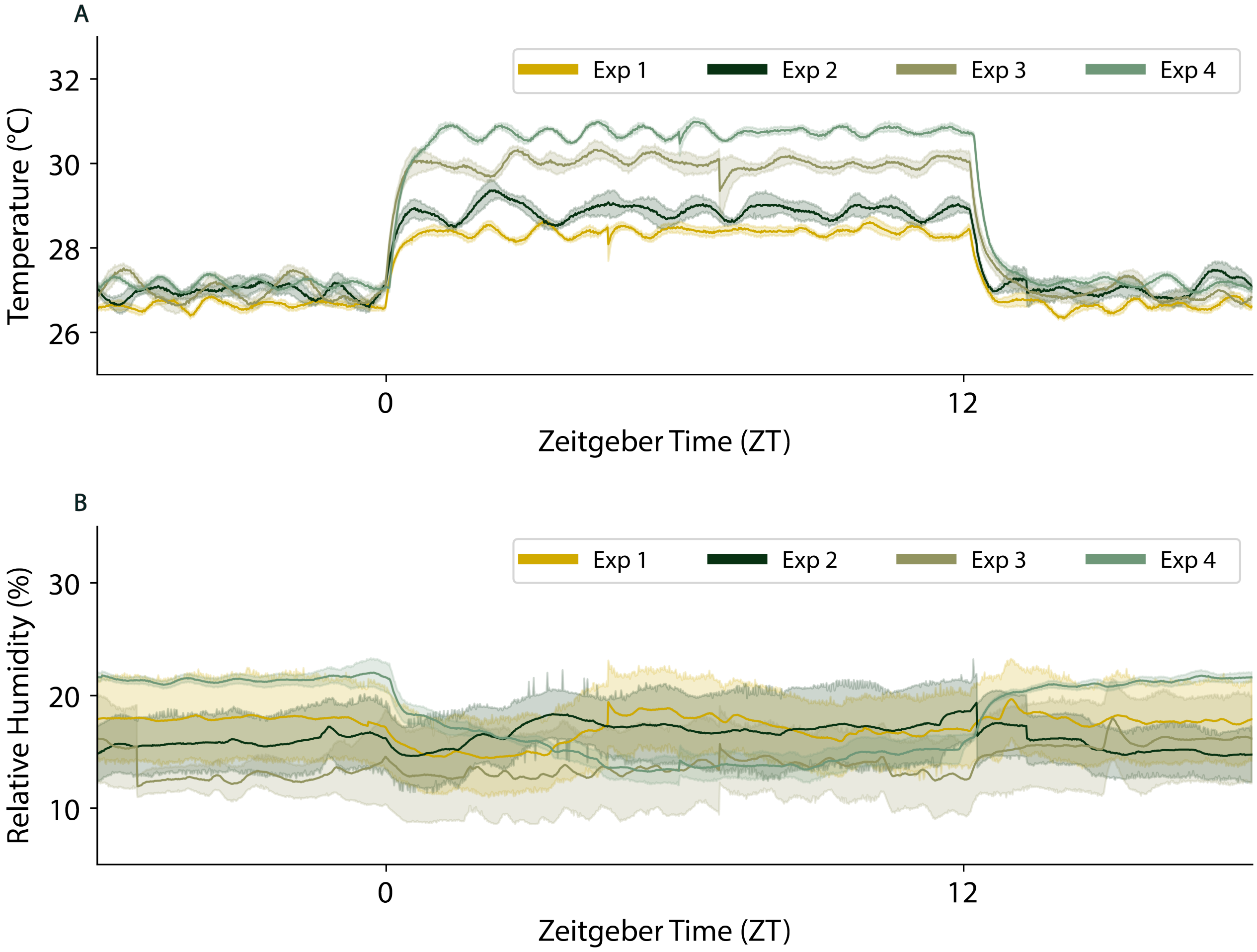

Figure S9. Line plots showing temperature and humidity measured throughout the experiments. All measurements were taken a 1 minute intervals and have been trimmed to only include points where at least one fly in the experiment is alive. **A** Line plot showing the variation in temperature across experiments and zeitgeber time. The shaded region is the standard error. **B** Line plot showing the variation in humidity across experiments and zeitgeber time. The shaded region is the standard error.
